# Diagnosis of pseudoxanthoma elasticum in a patient with discrete skin lesions^☆^

**DOI:** 10.1016/j.abd.2021.07.012

**Published:** 2023-04-20

**Authors:** Catalina Jahr, Valentina Vera, Roberto Bustos, José Contreras

**Affiliations:** a Department of Dermatology, Hospital Clínico de la Universidad de Chile, Santiago, Chile; b Department of Dermatology, Hospital Barros Luco Trudeau, Santiago, Chile

Dear Editor,

Pseudoxanthoma elasticum (PXE) is an autosomal recessive genetic metabolic condition characterized by aberrant calcification with the fragmentation of elastic fibers in the dermis, retina and tunica intima of arteries.^1^ We present the case of a patient with ocular alterations and slight cutaneous signs as a debut form of PXE, which was confirmed by skin biopsy.

A 33-year-old woman with a history of systemic lupus erythematosus was referred because an ocular fundus examination revealed angioid streaks associated with decreased foveolar brightness and diffuse pigmentary changes called “peau d’orange” (Fig. 1). Physical examination revealed two 2 mm whitish papules on the lateral cervical fold (Fig. 2). The patient was normotensive with peripheral and symmetrical pulses present. Calcium, phosphorus, and lipid profile values were normal. Histological examination of the cervical lesions revealed thickened and fragmented dermal elastic fibers. Von Kossa staining was positive for calcium deposition in elastic fibers (Fig. 3), confirming the diagnosis of PXE.

**Figure 1:**
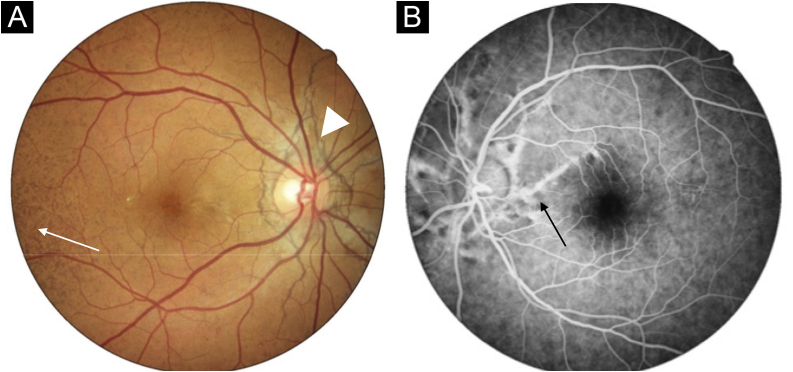
Fundus photograph of the right eye. Note the “peau d’orange” appearance of the fundus temporal to the macula (arrow) and barely visible angioid streaks radiate outwards from the optic disc giving the appearance of a “spider web” (arrowhead). Fluorescein angiography of the left eye demonstrated the angioid streaks with hyperfluorescent edges radiating from the optic nerve up to the midperiphery (arrow).

**Figure 2:**
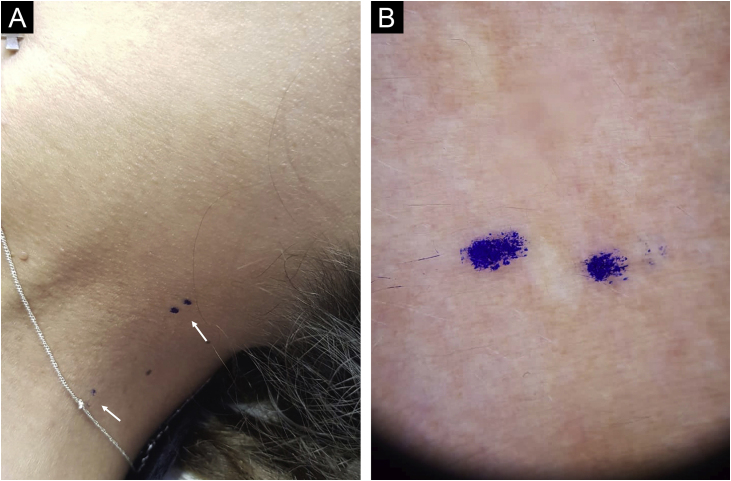
(A) Left cervicolateral skin area with two barely perceptible whitish papules, 2 mm in diameter, marked with a blue pencil (arrows). (B) Dermoscopy of one of the whitish papules marked with blue pencil.

**Figure 3:**
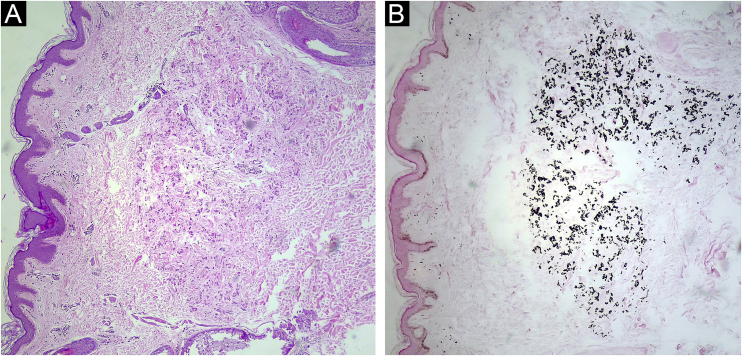
(A) Epidermis without alterations, dermis with increased and fragmented elastic fibers, which are thickened and with increased basophilia (Hematoxylin & eosin, 40×). (B) Von Kossa stain positive for calcium deposition in elastic fibers (Von Kossa, 40×).

The prevalence of PXE varies between 1/25,000 and 1/100,000, with female predominance. It is caused by a mutation in the ABCC6 gene, located on chromosome 16, which encodes a transmembrane transport protein MRP6.^2^ The pathophysiology involves reduced levels of the anti-mineralization factor inorganic pyrophosphate (PPi), dysfunctional extracellular calcium homeostasis, and ectopic mineralization of tissues rich in elastic fibers. There is a considerable phenotypic heterogeneity that could explain the late diagnosis of the clinical case presented.^3^

Although cutaneous findings usually represent the first clinical sign, they are typically subtle and not evident until the second or third decade.^3^ The first cutaneous sign is small, discrete, yellowish papules at the lateral side of the neck, axillae, antecubital and popliteal fossae. These papules coalesce to form plaques of corrugated and inelastic appearance.^2^, ^3^ Despite the innocuous appearance of these skin changes, they may reveal significant ocular and vascular involvement. Subsequently, it is associated with redundant skin in flexural folds, generally in the axillae and groin.^4^ In some patients with angioid streaks, a biopsy of healthy axillary or scar skin may reveal histological findings characteristic of PXE.^4^ Electron microscopy of the skin reveals bulky mineral deposits that disrupt and break elastic fibers in the mid-dermis.^2^

The first visible changes on funduscopy are pigment irregularities with a “peau d’orange” appearance that typically precedes angioid streaks by one to eight years. Angioid streaks originate from the optic disc and radiate outwards as brownish-grey irregular lines, histopathological findings show breaks of the calcified and thickened Bruch’s membrane. The latter predisposes to secondary choroidal neovascularization with the consequent risk of hemorrhage and blindness in the later stages of the disease.^5^ Although highly suggestive, angioid streaks have also been reported in patients with hemoglobinopathies, Paget’s disease, and Ehler-Danlos syndrome.^2^

Manifestations of vascular involvement include loss of peripheral pulses, claudication, hypertension, myocardial infarction and ischaemic or hemorrhagic strokes. These findings reflect the mineralization of the middle and intimal layers of small and medium-caliber arteries.^2^, ^3^

We present this case given the unusual clinical presentation and highlight the importance of cutaneous alterations in the diagnostic confirmation of this entity.

## Financial support

This research did not receive any specific grant from funding agencies in the public, commercial, or not-for-profit sectors.

## Authors’ contributions

Catalina Jahr: Approval of the final version of the manuscript; composition of the manuscript; collection, analysis, and interpretation of data; participation in the design of the study; critical review of the literature; critical review of the manuscript.

Valentina Vera: Approval of the final version of the manuscript; collection, analysis, and interpretation of data; critical review of the manuscript.

Roberto Bustos: Approval of the final version of the manuscript; collection, analysis, and interpretation of data; critical review of the manuscript.

José Contreras: Approval of the final version of the manuscript; collection, analysis, and interpretation of data; critical review of the manuscript.

## Conflicts of interest

None declared.
